# Urine TIMP2 × IGFBP7 increases 24 hours before severe AKI

**DOI:** 10.1186/cc13570

**Published:** 2014-03-17

**Authors:** M Ostermann, L Chawla, L Forni, J Kellum

**Affiliations:** 1 Guys & St Thomas Foundation Hospital, London, UK; 2 George Washington University, Washington, DC, USA; 3 Western Sussex Hospital, Worthing, UK; 4 University of Pittsburgh, PA, USA

## Introduction

We recently reported a 728-patient multicenter study (Sapphire) where a biomarker combination of tissue inhibitor of metalloproteinases-2 (TIMP-2) and insulin-like growth factor binding protein 7 (IGFBP7) were validated for risk stratification for moderate or severe acute kidney injury (AKI) KDIGO stage 2 and 3 [1].

## Methods

We subsequently selected two clinical cutoff values for the TIMP2 × IGFBP7 combination from the Sapphire dataset, one (0.3) with high sensitivity (89%) (specificity = 50%) and one (2.0) with high specificity (95%) (sensitivity = 42%) for the development of AKI KDIGO stage 2 and 3 within 12 hours of study enrolment. We examined the timing of change in TIMP2 × IGFBP7 relative to change in creatinine using the sign test.

## Results

**Figure 1:**
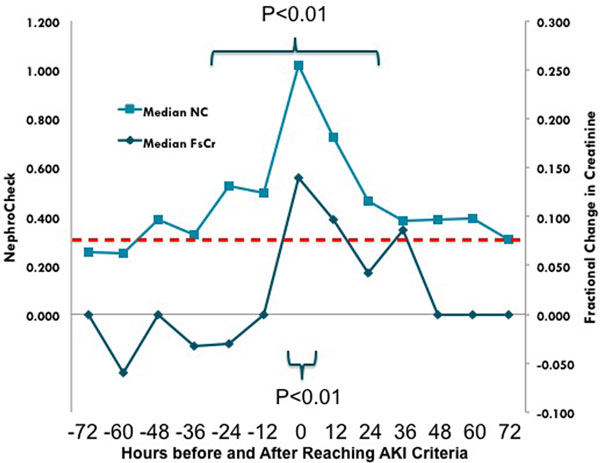
**NC, Nephrocheck (TIMP2 × IGFBP7); Cr, creatinine**.

TIMP2 × IGFBP7 results were available for 178 patients who developed AKI stage 2 or 3. The median TIMP2 × IGFBP7 result was significantly greater than the cutoff value of 0.3 from 24 hours before to 24 hours after AKI 2 or 3 (*P *< 0.01) (Figure 1). Conversely, median serum creatinine was not different from baseline prior to development of AKI 2 or 3.

## Conclusion

The TIMP2 × IGFBP7 biomarker combination identifies patients who ultimately develop moderate or severe AKI 24 hours earlier than serum creatinine.
